# Effect of piplartine and cinnamides on *Leishmania amazonensis*, *Plasmodium falciparum* and on peritoneal cells of Swiss mice

**DOI:** 10.1080/13880209.2017.1313870

**Published:** 2017-04-17

**Authors:** Keline Medeiros de Araújo-Vilges, Stefan Vilges de Oliveira, Shirley Claudino Pereira Couto, Harold Hilarion Fokoue, Gustavo Adolfo Sierra Romero, Massuo Jorge Kato, Luiz Antonio Soares Romeiro, José Roberto Souza Almeida Leite, Selma Aparecida Souza Kuckelhaus

**Affiliations:** a Laboratory of Cell Immunology, Faculty of Medicine, University of Brasilia Campus Darcy Ribeiro, Brasilia-DF, Brazil;; b Laboratory of Medical Parasitology and Vector Biology, Faculty of Medicine, University of Brasilia, Brasilia-DF, Brazil;; c Laboratory of Leishmaniasis, Nucleo of Tropical Medicine, Faculty of Medicine, University of Brasilia, Campus Darcy Ribeiro, Brasilia-DF, Brazil;; d Institute of Chemistry, University of São Paulo, São Paulo, SP, Brazil;; e Laboratory of Development and Therapeutic Innovation, Nucleo of Tropical Medicine, Faculty of Medicine, University of Brasilia, Campus Darcy Ribeiro, Brasilia-DF, Brazil;; f Laboratory of Morphology Faculty of Medicine, University of Brasilia Campus Darcy Ribeiro, Brasilia-DF, Brazil

**Keywords:** Piperlongumine, selectivity index, malaria, *Leishmaniasis*; *Piperaceae*

## Abstract

**Context:** Plants of the Piperaceae family produce piplartine that was used to synthesize the cinnamides.

**Objective:** To assess the effects of piplartine (**1**) and cinnamides (**2–5**) against the protozoa responsible for malaria and leishmaniasis, and peritoneal cells of Swiss mice.

**Materials and methods:** Cultures of *Leishmania amazonensis*, *Plasmodium falciparum*-infected erythrocytes, and peritoneal cells were incubated, in triplicate, with different concentrations of the compounds (0 to 256 μg/mL). The inhibitory concentration (IC_50_) in *L. amazonensis* and cytotoxic concentration (CC_50_) in peritoneal cell were assessed by the MTT method after 6 h of incubation, while the IC_50_ for *P. falciparum*-infected erythrocytes was determined by optical microscopy after 48 or 72 h of incubation; the Selectivity Index (SI) was calculated by CC_50_/IC_50_.

**Results:** All compounds inhibited the growth of microorganisms, being more effective against *P. falciparum* after 72 h of incubation, especially for the compounds **1** (IC_50_ = 3.2 μg/mL) and **5** (IC_50_ = 6.6 μg/mL), than to *L. amazonensis* (compound **1 **=** **179.0 μg/mL; compound **5 **=** **106.0 μg/mL). Despite all compounds reducing the viability of peritoneal cells, the SI were <10 to *L. amazonensis*, whereas in the cultures of *P. falciparum* the SI >10 for the piplartine (>37.4) and cinnamides **4** (>10.7) and **5** (= 38.4).

**Discussion and conclusion:** The potential of piplartine and cinnamides **4** and **5** in the treatment of malaria suggest further pre-clinical studies to evaluate their effects in murine malaria and to determine their mechanisms in cells of the immune system.

## Introduction

Infectious diseases such as malaria and leishmaniasis cause thousands of deaths per year in the world, especially in tropical countries where warm and humid climate favours the dissemination of vectors (Alvar et al. 2012; Braz et al. 2013; Burrows et al. 2014).

Malaria is caused by protozoa of the genus *Plasmodium*, *P. falciparum* being the species responsible for the largest number of severe cases of the disease causing impairment of the brain, lungs, and kidneys (Gomes et al. 2011) with mortality reaching 50% of the cases (Oliveira-Ferreira et al. 2010). Leishmaniasis are caused by various protozoa species of *Leishmania* genus (Schonian et al. 2010, 2011) that manifest themselves clinically in cutaneous, mucocutaneous, and visceral forms (Pham et al. 2013). Together, these diseases are responsible for hundreds of thousands of deaths each year (Handler et al. 2015; World Health Organization 2016).

Currently, the treatment of malaria is based on the combination of artemisinin derivatives (Beteck et al. 2014). However, the development of resistance mechanisms by infectious agents has hindered disease control (Clark et al. 2014). Similarly, for the control of leishmaniasis, the difficulties are based on the low efficacy and high toxicity of antimony derivatives and amphotericin B, including its liposomal formulation and high cost (Monzote 2009). Together, these difficulties stimulate the search for new therapeutic agents.

In Northeast Brazil, plants of the Piperaceae family are known to produce compounds with microbicide activities (Parmar et al. 1997). Among these, piplartine (3,4,5-trimethoxycinnamoyl-*N*-dihydropyridin-2-one), also known as piperlongumine, found in the *Piper* genus (Bezerra et al. 2013) presents anti-inflammatory, antitumor, antifungal and antiparasitic effects (Raj et al. 2011; Moraes et al. 2011; Adams et al. 2012; Rao et al. 2012). Given these findings, piplartine has been used as a scaffold for synthesis of other amides, such as cinnamides, using the molecular simplification strategy to establish relation between chemical structures and their biological effects (Fokoue 2015).

The effectiveness of a bioactive compound is characterized by its microbicidal effect and toxicity to mammalian cells (Houghton et al. 2007). Considering the difficulties in controlling malaria and leishmaniasis by the low availability and high toxicity of drugs, in addition to the acquisition of resistance, our study aimed to determine the antiparasitic effect of piplartine and four cinnamides in cultures of *L. amazonensis* and *P. falciparum*, as well their selectivity index (SI), after the determination of their toxicity to peritoneal cells of mice, for future biomedical and biotechnological applications.

## Material and methods

### Piplartine and cinnamides

Piplartine (**1**) was obtained from the roots of *Piper tuberculatum* Jacq (Piperaceae) collected 4 May 2015 from a plant growing at the garden of Institute of Chemistry (University of São Paulo). The identification was carried out by Dr. Elsie F. Guimarães and a voucher was (Kato-0169) was deposited at Herbarium of the Jardim Botânico of Rio de Janeiro, Brazil. The roots were dried at 60 °C for 48 h, then ground 105 g of a powder was extracted four times with a dichloromethane:methanol (2:1; 400 mL). The extract was filtered and concentrated in a rota evaporator. The crude extract was submitted to recrystallization using ethyl acetate and methanol yielding pure piplartine (150 mg) (Cotinguiba et al. 2009).

The secondary (**2**, **3**) and tertiary (**4**, **5**) cinnamides were synthesized by adding triethylamine (3 equiv.) and amine (*N*-pentylamine and morpholine) to a solution of acid chloride (1 equiv) in CH_2_Cl_2_. In order to prepare the acid chloride, a solution of (*E*)-3,4,5-trimethoxycinnamic acid and (*E*)-3,4-dimethoxycinnamic acid (1 equiv.), both prepared by Knoevenagel condensation using 3,4,5-trimethobenzaldehyde and 3,4-dimethobenzaldehyde and malonic acid, in dry THF (10 mL), kept under nitrogen atmosphere, oxalyl chloride (5 equiv.) was added drop wise and stirred at room temperature for 5–6 h. The excess of oxalyl chloride was then removed under reduced pressure yielding the corresponding acid chloride (Fokoue 2015). The reaction mixture was stirred overnight at room temperature and then quenched with saturated aqueous NH_4_Cl, and extracted with CH_2_Cl_2_ (3 times). The combined organic phases were washed with brine and dried over MgSO_4_. After filtration and concentration, the residue was purified by flash chromatography to provide the desired amide (Adams et al. 2012).

**(1) Piplartine**: **1-[(*E*)-3-(3,4,5-trimethoxyphenyl)prop-2-enoyl]-2,3-dihydropyridin-6-one** (CAS: 20069-09-4 and PubChem CID: 637858): ^1 ^H NMR (300 MHz; CDCl_3_): δ 7.68 (*d*, 15.6 Hz, 1 H, H-2), 7.43 (*d*, 15.6 Hz, 1 H, H-3), 6.95 (*m*, 1 H, H-2′), 6.81 (*s*, 2 H, H-5, H-9), 6.04 (*dt*, 9.9 and 1.8 Hz, 1 H, H-3′), 4.05 (*t*, 6.5, 2 H, H-5′), 3.89 (*s*, 6 H, OMe-6 and 8), 3.88 (*s*, 3 H, OMe-7), 2.50 (*m*, 2 H, H-4′).^13 ^C NMR (75 MHz, CDCl_3_): δ 168.70 (C-1), 165.76 (C-1′), 153.30 (C-6, C-8), 145.50 (C-3′), 143.70 (C-4), 139.90 (C-7), 130.60 (C-3), 125.70 (C-2′), 121.00 (C-2), 105.40 (C-5, C-9), 60.89 (OMe-7), 56.11 (OMe-6 and 8), 41.62 (C-5′), 24.70 (C-4′). EI-MS (*m/z*) (%): 317 (90, M^+^), 274 (32), 221 (100), 190 (32). ESI-MS: [M + H]^+^ (*m/z*) calculated: 318.1336; found 318.1337.

**(2) (E)-1-morpholin-4-yl-3-(3,4-dimethoxyphenyl)prop-2-en-1-one** (CAS: 1215221-17-2 and PubChem CID: 6438283): ^1 ^H NMR (200 MHz, CDCl_3_): δ 7.62 (*d*, 15.4 Hz, 1H, H-3), 6.74 (*s*, 2H, H5, H-9), 6.72 (*d*, 15.4 Hz, 1H, H-2), 3.90 (*s*, 6H, OMe-6 and 8), 3.88 (*s*, 3H, OMe-7), 3.73 (*s*, 8H, H-1′, H-2′, H-4′, H-5′). ^13^C NMR (50 MHz, CDCl_3_): δ 165.47 (C-1), 153.36 (C-6, C-8), 143.26 (C-3), 139.67 (C-7), 130.61 (C-4), 115.67 (C-2), 105.00 (C-5, C-9), 66,80 (C-2′, C-4′), 60.89 (OMe-7), 56.15 (OMe-6 and 8), 45,56 (C-1′), 42,36 (C-5′). EI-MS (*m/z*) (%): 307 (50, M^+^), 222 (60), 221 (100), 190 (25). ESI-MS: [M+H]^+^ (*m/z*) calculated: 308.1492, found: 308.1496. 

**(3) (E)-1-morpholin-4-yl-3-(3,4,5-trimethoxyphenyl)prop-2-en-1-one** (CAS: 1703-34-06 and PubChem CID: 438283): ^1^H NMR (200 MHz, CDCl_3_): δ 7.66 (*d*, 15.4 Hz, 1H, H-3), 7.14 (*dd*, 8.4 and 2.0 Hz, 1H, H-8), 7.04 (*d*, 2.0 Hz, 1H, H-5), 6.87 (*d*, 8.4 Hz, 1H, H-9), 6.71 (*d*, 15.4 Hz, 1H, H-2), 3.93 (*s*, 3H, OMe-6), 3.92 (*s*, 3H, OMe-7), 3.73 (*s*, 8H, H-1′, H-2′, H-4′, H-5′). ^13^C NMR (50 MHz, CDCl_3_): δ 165.77 (C-1), 150.63 (C-6), 149.10 (C-7), 143.20 (C-3), 128.07 (C-4), 121.86 (C-9), 114.16 (C-2), 111.07 (C-8), 109.86 (C-5), 66.83 (C-2′, C-4′), 55.91 (OMe-6 and 7), 45.61 (C-1′), 42.69 (C-5′). EI-MS (*m/z*) (%): 277 (31.M^+^), 192 (23), 191 (100). ESI-MS: [M+H]^+^ (*m/z*) calculated: 278.1387, found: 278.1389.

**(4) (E)-3-(3,4-dimethoxyphenyl)-N-pentylprop-2-enamide** (CAS: 574007-48-0 and 19173974): ^1^H NMR (200 MHz, CDCl_3_): δ 7.54 (*d*, 15.6 Hz, 1H, H-3), 6.73 (*s*, 2H, H5, H-9), 6.32 (*d*, 15.6 Hz, 1H, H-2), 3.88 (*s*, 6H, OMe-6 and 8), 3.87 (*s*, 3H, OMe-7), 3.39 (*t*, 6.7 Hz, 2H, H-1′), 1.58 (*m*, 2H, H-2′), 1,35 (*m*, 4H, H3′, H-4′), 0.91 (*t*, 7.4 Hz, 3H, H-5′). ^13^C NMR (50 MHz, CDCl_3_): 165.72 (C-1), 153.36 (C-6, C-8), 140.73 (C-3), 139.50 (C-7), 130.45 (C-4), 120.15 (C-2), 104.88 (C-5, C-9), 60.91 (OMe-7), 56.10 (OMe-6 and 8), 39.75 (C-1′), 29.35 (C-2′), 29.08 (C-3′), 22.35 (C-4′), 13.95 (C-5′). EI-MS (*m/z*) (%): 307 (85, M^+^), 236 (45), 222 (100), 221 (95), 190 (30), 181 (55). ESI-MS: [M+H]^+^ (*m/z*) calculated: 308.1856, found: 308.1859.

**(5) (E)-3-(3,4,5-trimethoxyphenyl)-N-pentylprop-2-enamide** (CAS: 467247-64-9 and PubChem CID: 5856629 ): ^1^H NMR (200 MHz, CDCl_3_): δ 7.56 (*d*, 15.5 Hz, 1H, H-3), 7.11-7.06 (*dd*, 8.4 and 1.8 Hz, 1H, H-9), 7.02 (*d*, 1.8 Hz, 1H, H-5) 6.85 (*d*, 8.4 Hz, 1H, H-8), 6.26 (*d*, 15.5 Hz, 1H, H-2), 3.91 (*s*, 6H, OMe-6 and 7), 3.38 (*m*, 2H, H-1′), 1.61-1.54 (*m*, 2H, H-2′), 1.38-1.32 (*m*, 4H, H-3′, H-4′), 0.91 (*t*, 7.2 Hz; 3H, H-5′). ^13^C NMR (50 MHz, CDCl_3_): δ 166.03 (C-1), 150.51 (C-6), 149.11 (C-7), 140.66 (C-3), 127.87 (C-4), 121.85 (C-9), 118.68 (C-2); 111.08 (C-8), 109.66 (C-5), 55.94 (OMe-6), 55.86 (OMe-7), 39.73 (C-1′), 29.41 (C-2′), 29.10 (C-3′), 22.37 (C-4′), 13.98 (C-5′). EI-MS (*m/z*) (%): 277 (43. M^+^), 206 (35), 192 (40), 191 (100), 151 (45). ESI-MS: [M+H]^+^ (*m/z*) calculated: 278.1751, found: 278.1758.

### Microorganisms

#### Leishmania amazonensis

The MHOM/BR/pH8 strain was transferred to the NNN solid medium (Novy-MacNeal-Nicolle) for 48 h at 24 °C. Then the promastigote forms were transferred and cultured in RPMI 1640 medium supplemented with 10% of inactivated fetal calf serum (Sigma-Aldrich, St. Louis, MO) and gentamycin (40 mg/mL) (Schering-Plough, Sao Paulo, Brazil) until the parasites reached the log phase of growth.

#### Plasmodium falciparum

The Pf/Unb169 strain, kept under cryopreservation was culture together with O^+^ erythrocytes in RPMI 1640 medium, supplemented with hypoxanthine (0.25 mg/dL), 10% of human serum, and gentamicin (40 mg/mL), at 37 °C in air with 5% CO_2_. For the assays, a suspension with 3% of hematocrit and 0.6% of infected erythrocytes was prepared.

### Animals

To obtain peritoneal cells, six adult Swiss mice (male and female), weighing 30 ± 6 g, were used. During the experiments, the animals were kept in the Faculty of Medicine, University of Brasilia under ambient temperature, light/dark cycle of 12 h, fed with balanced diet and water *ad libitum*. The study was approved by the Ethics Committee on Animal Use (CEUA) of the Institute of Biological Sciences at University of Brasilia (Doc. no. 22199/2014).

### Determination of inhibitory and cytotoxic concentrations of piplartine and cinnamides

To assess inhibitory or cytotoxic concentrations of the compounds (**1–5**), able to eliminate 50% of the cells in the cultures of *L. amazonensis* (IC_50_) and cytotoxic concentrations (CC_50_) in the cultures of peritoneal cell, MTT [3-(4,5-dimethylthiazol-2-yl)-2,5-diphenyltetrazolium bromide] (Sigma-Aldrich) method was used. This method evaluates the cell viability by the activity of mitochondrial succinate dehydrogenase enzyme that cleaves the tetrazolium salt to form formazan crystals in living cells. For the cultures of *P. falciparum*-infected erythrocytes, the IC_50_ were determined by optical microscopy. The concentrations for the compounds tested were calculated in μg/mL (ranging from 0 to 256 μg/mL), as well their equivalent in g/mol, being: **1** (0 to 806.7 μM), **2** (0 to 833.0 μM), **3** (0 to 923.0 μM), **4** (0 to 832.8 μM) and **5** (0 to 923.1 μM).

#### Cultures of *L. amazonensis*

The assay was performed with promastigotes forms of *L. amazonensis* (10^6^/well/200 μL) incubated for 2 h at 26 °C in 96 wells plate, in triplicate, in RPMI 1640 medium, supplemented with 10% foetal bovine serum, in the presence or not of various concentrations (0 to 256 μg/mL) of the compounds (**1–5**); 4 μg/mL of antimoniate of *N*-metilglucamine (Glucantime^®^ Rhodia Pharma, São Paulo, Brazil) (Brand et al. 2006) or RPMI 1640 medium as positive or negative control, respectively, were used. Then, 10 μL of MTT solution (5 mg/mL) was added to all wells and the cultures were re-incubated at the same condition for 4 h, but protected from light. For the solubilization of the formazan crystals 50 μL DMSO was added to each well; the absorbance was obtained in a spectrophotometer (Spectra Max ® Plus 384, Molecular Devices, Sunnyvale, CA) with 570 nm. The results were expressed in percentage.

#### Cultures of *P. falciparum*

A suspension of O^+^ human erythrocytes (3% hematocrit and 0.6% of infected erythrocytes) was incubated at 37 °C with 5% of CO_2_ in air, for 48 or 72 h in RPMI 1640 medium supplemented with 10% human serum, in triplicate, in 96 wells plate, with different concentrations (0 to 256 μg/mL) of compounds (**1**–**5**). As positive control, infected erythrocytes were incubated with 8 ng/mL of artesunate (Artemix Ativus, São Paulo, Brazil) and, as negative control, they were incubated exclusively with the diluent (complete RPMI 1640 medium); 50% of the medium, for the cultures incubated for 72 h, was exchanged after 48 h, at the same concentrations as before. After that, 150 μL of the supernatant of the cultures were discarded, and a drop of suspension distended onto slides for microscopy, dried and fixed with methanol, was stained for 15 min with 10% of Giemsa solution (PBS, pH 6.9). The erythrocytes (1000 cells/slide) were analyzed by a single observer with optical microscopy and the results were expressed in percentage of infected cells.

### Determination of cytotoxicity of piplartine and derivatives

Initially, cells obtained by washing the peritoneal cavity of the mice with 10 mL of PBS pH 7.2 at 4 °C, centrifuged at 400 *g* for 10 min and suspended with 1 mL of RPMI 1640 medium were quantified at Neubauer chamber with 0.05% of nigrosin solution. Then, a suspension containing 2 × 10^5^ cells/well (>99% macrophages) (Muniz-Junqueira et al. 1997) was distributed in triplicate in 96 wells plate, in the presence of different concentrations (0 to 256 μg/mL) of amides (**1**–**5**) and incubated for 2 h at 37 °C with 5% of CO_2_ in air; RPMI 1640 medium without cells was used as control. After that, 10 μL of a MTT solution (5 mg/mL) to all wells were added and, the plate was re-incubated protected from light for 4 h. The formazan crystals were solubilized with 50 μL of DMSO/well and the absorbance obtained in spectrophotometer (Spectra Max® Plus 384) with 550 nm. The results expressed in percentage of viable cells.

### Determination of the SI

The SI for each amide (**1–5**) was obtained by dividing the concentration able to eliminate 50% of peritoneal cells (CC_50_) by the concentration able to inhibit the growth of 50% of the protozoa (IC_50_). Compounds which presented SI greater than 10 were considered potentially microbicides and safe for the host cells (Bézivin et al. 2003).

### Statistical analysis

**Figure 1.**
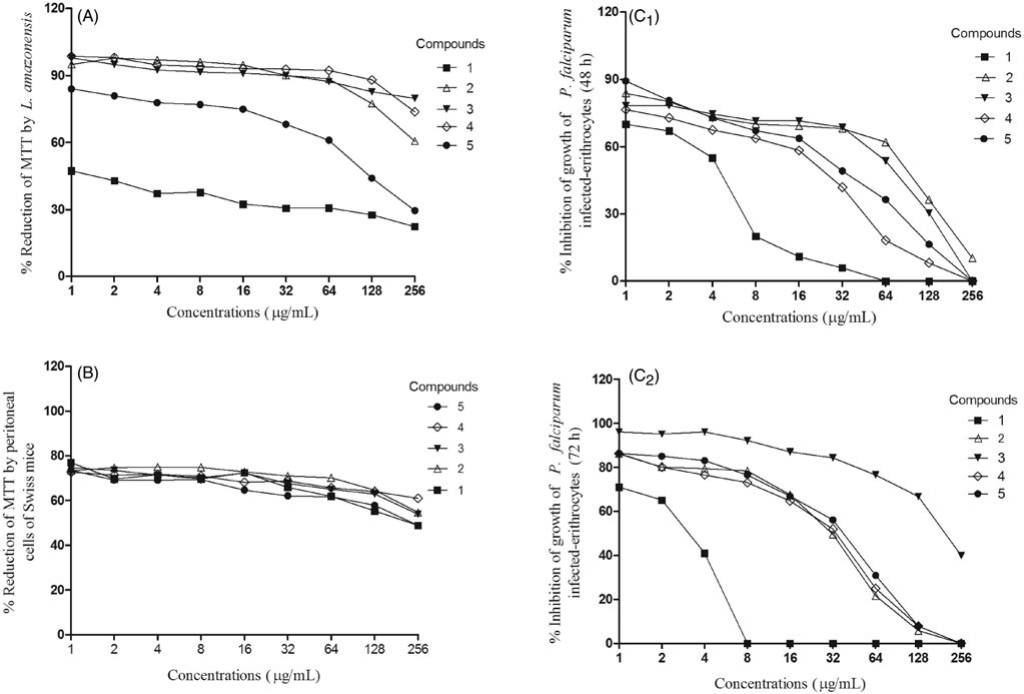
Percentage of MTT reduction by cultures of *L. amazonensis* (A) and peritoneal cells of Swiss mice (B) after 6 h of incubation, and inhibition of growth of *P. falciparum* after 48 (C_1_) or 72 (C_2_) h of incubation.

The normality of the variables was analyzed employing the Kolmogorov–Smirnov test and, considering the normal distribution, the paired *t*-test to compare two dependent samples was used. The curves of the Figure 1 were fit using nonlinear regression (Exponential/three phase decay) with *r*^2^ = 0.987 ± 0.02. The PRISM ® Software Package program (GraphPad, USA, 2005) was used to analyze and represent the results. Differences were considered significant at *p* value <0.05.

## Results

### Effects of piplartine and cinnamides in the cultures

#### Leishmania amazonensis

The incubation of piplartine (**1**) or the cinnamides (**2**–**5**) for 6 h inhibited the growth of *L. amazonensis* promastigotes in a dose-dependent pattern, when compared to the control (Pared *t-*test; *p* < 0.05); *N*-metilglucamine was able to inhibit 55.4% of the parasites after 6 h of incubation. The viable parasites as well as the estimated inhibitory concentration able of inhibiting 50% of parasites (IC_50_) are shown in the Table 1 and Figure 1(A).

**Table 1.** Chemical structure of piplartine (**1**) and cinnamides (**2–5**), and concentrations able to inhibit 50% of the cells (IC_50_), *L. amazonensis*, *P. falciparum* and peritoneal cells of Swiss mice.

		Selectivity Index (SI) = CC_50_/ IC_50_			
		Estimated IC_50_: μM (μg/mL)			Estimated CC_50_: μM (μg/mL)
		*L. amazonensis*	*P. falciparum*		Peritoneal cell
Compounds	Chemical structure Molar Mass (g/mol)	6 h	48 h	72 h	6 h
**1**	317.34	564.1 (179.0) SI = 1.3	19.5 (6.2) SI = 37.4^a^	10.1 (3.2) SI = 72.5^a^	731.1 (232.0)
**2**	307.34	1,099.7 (338.0) SI = 0.7	325.4 (100.0) SI = 2.6	126.9 (39.0) SI = 6.8	868.7 (267.0)
**3**	277.32	2,141.9 (594.0) SI = 0.4	334.9 (92.9) SI = 2.9	623.8 (173.0) SI = 1.5	988.2 (274.0)
**4**	307.38	1,411.9 (434.0) SI = 1.0	79.1 (24.3) SI = 18.3^a^	134.7 (41.4) SI = 10.7^a^	1,447.7 (445.0)
**5**	277.36	382.2 (106.0) SI = 2.4	101.7 (28.2) SI = 9.0	23.8 (6.6) SI = 38.4^a^	915.8 (254.0)

a Compounds potentially microbicides and safe for the host cells, once the SI was greater than 10.

#### Plasmodium falciparum

The results obtained from the cultures of *P. falciparum*-infected erythrocytes has demonstrated that incubation for 48 or 72 h with piplartine (**1**) was able to inhibit 100% of growth of parasites with 64 or 8 μg/mL, respectively, when compared to the control (Pared *t*-test; *p* < 0.05). Piplartine (**1**) and the other amides (**2**–**5**) inhibited the growth of *P. falciparum* in a dose-dependent pattern, being that inhibition of 100% of the parasites was reached with 8 μg/mL of piplartine for 72 h of incubation (Table 1, Figure 1 C1, C_2_); artesunate was able to inhibit 79.8% or 90.0% of the parasites after 48 or 72 h of incubation.

### Peritoneal cells of Swiss mice

The assays to evaluate possible cytotoxic effects of piplartine (**1**) and cinnamides (**2**–**5**) to peritoneal cells showed that incubation for 6 h with different concentrations of the compounds were able to reduce cell viability, when compared to the control, in a dose-dependent pattern, being the piplartine more toxic (CC_50_ = 232 μg/mL) and compound 4 less toxic to the cells (CC_50_ = 445 μg/mL) (Pared *t*-test; *p* < 0.05) (Table 1, Figure 1(B)); the compounds incubated in RPMI without cells were not able to reduce MTT salt, once the absorbance were similar to the culture medium.

### Selectivity index

The SI obtained for promastigotes forms of *L. amazonensis* were lower than 10 for all compounds (**1**–**5**), whereas for cultures of *P. falciparum* the SI were higher than 10 for the compounds **1** and **4** incubated for 48 or 72 h and, for the compound **5** incubated for 72 h (Table 1).

## Discussion

As previously reported, the difficulties of controlling malaria and leishmaniasis by the low availability and high toxicity of drugs, in addition to the acquisition of resistance is a reason to search for new compounds. Facing these difficulties, and due to the potential of piplartine as an anti-protozoa agent, our study has determined the effects of piplartine and four cinnamides in the cultures of protozoa (*L. amazonensis* and *P. falciparum*) and peritoneal cells of mice to assess the SI of these compounds.

Piplartine and cinnamides were able to reduce the growth of promastigotes forms of *L. amazonensis* in a dose-dependent pattern, the lowest being IC_50_ obtained for the compound **5** (106 μg/mL) and the highest for compound **3** (594 μg/mL). The leishmanicidal potential of amides derived from the genus *Piper* was also observed in cultures of promastigotes of *L. donovani*, as well, in the reduction of parasitemia of hamsters with visceral leishmaniasis (Bodiwala et al. 2007). However, despite anti-protozoa results for piplartine and cinnamides, the lower SI(SI <10; Table 1) obtained in this study, indicate that molecular modifications should be considered in future studies.

The results for the cultures of *P. falciparum* showed inhibition of growth for the piplartine and cinnamides in a dose-dependent pattern, as observed for compounds derived from benzoic acids of the genus *Piper* (Rahman et al. 1999; Flores et al. 2008). In our study, the results showed that piplartine was most effective against *P. falciparum*, in comparison to the others cinnamides (**2**–**5**), exhibiting the lowest IC_50_ after 72 h of incubation, which, coupled with the low potential cytotoxic obtained for peritoneal cells, resulted in a selectivity 72.5 times greater to the *P. falciparum* than to peritoneal cells (Table 1).

The evaluation of toxicity to peritoneal/mammalian cells was essential to determine the safety of using piplartine and cinnamides in future studies. The CC_50_ of 232 μg/mL for piplartine indicated lower selectivity to the peritoneal cells in comparison to the *P. falciparum* (IC_50_/72 h = 3.2 μg/mL) or *L. amazonensis* (IC_50_ = 179 μg/mL).

In a previous screening, piplartine (**1**) showed an inhibitory effect on the growth of *L. amazonensis* promastigotes 4.07 fold superior to its piperidine analogue (IC_50_ 2297.9 μM) (data not shown), which was generated by the substitution of the 5,6-dihydropyridin-2 (1 H)-one subunity by piperidine. This found pointed out to the contribution of Michael acceptor system in the inhibitory activity of the compounds. The bioisosteric replacement of the methylene group in the piperidine ring by an oxygen atom in the morpholine derivative (**2**) increased the activity profile (2.09-fold) suggesting the relevance of hydrophilic interactions. In turn, molecular simplification of 3,4,5-trimethoxycinnamoyl group (TM) by 3,4-dimethoxycinnamoyl group (DM) decreased the activity of compound (**3**), (1.95-fold), reinforcing the presence of hydrogen bond acceptors (ALH) and dipole-dipole or ion-dipole interactions of these compounds. Then, the opening of the piperidine ring was explored in order to compare the contribution of cyclic and acyclic hydrophobic subunits. In this sense, the secondary cinnamides containing *N*-pentyl group (**4** and **5**) were designed aiming at evaluating the activity of the hydrophilic derivatives via interactions with hydrogen bonding donor group (HBD) and ion-dipole interactions. Thus, the 3,4,5-trimethoxycinnamide (**4**) showed inhibitory activity 1.63 fold that found for piperidine derivative. Unlike of the observed results for morpholine derivatives (**2**) and (**3**), the 3,4-dimethoxycinnamide (**5**) showed the best profile of all the compounds evaluated. This result indicates that the presence of DLH hydrophilic group in the subunit cynnamide has reversed the contribution of methoxy-substituted aromatic groups, in which the DM subunit interacts more significantly than the TM subunit. In addition, the *N*-pentylamide (**5**) showed an increase in the activity (6.02-fold) compared to the piperidine analogue, suggesting that the presence of the biophoric conformationally free *N*-pentyl group, that confers dual hydrophilic and hydrophobic profile of the subunity, strengthens the hydrophobic interactions at the same time that contribute to the ion-dipolo and Keesom forces interactions.

Considering the experiments for cultures of *P. falciparum*-infected erythrocytes the results showed that after 48 h, piplartine (**1**) presented the best inhibitory activity followed by the secondary *N*-pentylcinnamides (**4**) and (**5**) (TM subunit was 1.28-fold better than DM), while the morpholine derivatives (**2**) and (**3**) showed similar profile. These results point out the relevance of the molecular characteristic above mentioned, where the contribution of the conformational freedom of the hydrophobic moiety combined to the hydrophilic HBD group impact positively on the inhibitory effect on the growth of the parasite.

The results from 72 h of incubation showed that piplartine (**1**) was the best compound followed by the secondary N-pentylcinnamide (**5**), while DM morpholine was the less effective compound (**3**), and the two TM derivatives, morpholine (**2**) and the secondary *N*-pentylcinnamide (**4**), showed similar inhibitory activity. These results are insufficient to identify molecular characteristics related to the found activities, and suggest exploring possible physical-chemistry descriptors or even to increase the number of compounds in order to establish the SAR and to validate the rational design of this class of compounds.

Although the mechanism of action of piplartine is unclear, it has been reported that this compound is able to inhibit proliferative processes and to activate apoptotic events in neoplastic cells (Bodiwala et al. 2007; Cotinguiba et al. 2009; Bezerra et al. 2013), possibly causing damage to DNA by inhibiting the enzymatic activity of topoisomerase II (Bezerra et al. 2013). Another mechanism of cell death is related to the decreasing in the metabolism and in the production of reactive oxygen species, as observed for normal dendritic cells (Xiao et al. 2016). Therefore, considering that the piplartine presented toxicity for the three biological systems studied, it is possible that the same reported microbicidal mechanisms have acted in a dose-dependent pattern. However, the lower toxicity of piplartine coming from peritoneal cells could reflect the complexity of these cells in comparison to the protozoa.

Other aspect that could be related to the effects of piplartine and cinnamides refers to the regulation of cellular processes mediated by the ubiquitin-proteasome system (UPS). This is a large protein complex of eukaryotic cells that act in cellular pathways for the degradation of proteins in the cytosol and, is involved in cell proliferation, apoptosis and antigen presentation (Glickman & Ciechanover 2002; Muñoz et al. 2015). The blocking of the proteasomic activity by the lactacystin reduced the growth of *L. chagasi* showing the importance of this complex in basic cellular processes of protozoa (Silva-Jardim et al. 2004). The findings of Jarvius et al. (2013) showed that piplartine was able to inhibit the ubiquitin-proteasome system (UPS) and to induce the production of reactive oxygen species by tumour cells. Considering these studies, it is possible to assume that the microbicidal effect of piplartine and cinnamides in the cultures of protozoa may be related to the inhibition of the UPS resulting in a lower cell metabolism and activation of apoptotic pathways.

Especially to the *P. falciparum*, it is speculated that the piplartine and cinnamides could act in its survival mechanisms. It is known that the surviving of *Plasmodium* into erythrocytes is possible due to the ability of parasites to polymerize, via plasmepsin and falcipain, the heme radical that is generated from the enzymatic digestion of hemoglobin within a parasitophorous vacuoles (Olliaro & Goldberg 1995), thereby preventing the toxicity mediated by reactive oxygen species (Ginsburg et al. 1999; Sullivan 2002). Then, it is possible that the piplartine and cinnamides could affect the enzymatic activity related to the digestion of hemoglobin by the parasites, preventing the supply of nutrients and heme polymerization and, consequently, their exposure to toxicity mediated by iron; this aspect may have favoured the higher toxicity of the compounds for the *Plasmodium*.

Our results indicate the potential of piplartine to treat malaria, including its inhibitory effects in the production of reactive oxygen species and in the cellular pathways related to the production of cytokines, JNK and NF-κB (Xiao et al. 2016), that could minimize the effects observed in the immunopathogenesis of severe malaria (Muniz-Junqueira 2007). From the high SI obtained to *P. falciparum* (>10) it is suggested other pre-clinical studies with piplartine (**1**) and cinnamides **4** [(*E*)-*1*- morpholin-4-yl-3-(3,4-dimethoxyphenyl)prop-2-en-1-one] and **5 [**(E)-1-morpholin-4-yl-3-(3,4,5-trimethoxyphenyl)prop-2-en-1-one] to evaluate their effects in the treatment of murine malaria and, to determine their mechanisms of action in the cells of the immune system.

This study has shown the potential antiprotozoal activity of amides with low toxicity to host cells and demonstrated that piplartine and cinnamides **4** and **5** are promising alternatives in the treatment of malaria.
